# Thermal, Mechanical, Viscoelastic and Morphological Properties of Poly(lactic acid) based Biocomposites with Potato Pulp Powder Treated with Waxes

**DOI:** 10.3390/ma12060990

**Published:** 2019-03-26

**Authors:** Maria Cristina Righetti, Patrizia Cinelli, Norma Mallegni, Carlo Andrea Massa, Laura Aliotta, Andrea Lazzeri

**Affiliations:** 1 CNR—IPCF, National Research Council—Institute for Chemical and Physical Processes, Via Moruzzi 1, 56124 Pisa, Italy; patrizia.cinelli@unipi.it (P.C.); norma.mallegni@pi.ipcf.cnr.it (N.M.); carlo.andrea.massa@pi.ipcf.cnr.it (C.A.M.); andrea.lazzeri@unipi.it (A.L.); 2 Department of Civil and Industrial Engineering, University of Pisa, Largo Lucio Lazzarino 1, 56122 Pisa, Italy; laura.aliotta@dici.unipi.it

**Keywords:** bio-based polymers, natural fibers, biomass, biocomposites, fiber/matrix adhesion, compatibilizer

## Abstract

The thermal, mechanical and viscoelastic properties of biocomposites of poly(lactic acid) (PLA) with 20 wt.% of potato pulp powder were investigated. The potato pulp powder utilized is a byproduct from the production and extraction of starch. The results showed that the potato pulp powder does not act as reinforcement, but as filler for PLA, due to an unfavorable aspect ratio and the irregular shape of the particles. In order to improve the mechanical response of the PLA/potato pulp powder biocomposites, surface treatment of the potato pulp particles with bio-based and petroleum-based waxes was investigated. This treatment was found to improve the properties of the biocomposites, enhancing the adhesion between the PLA based polymeric matrix and the potato pulp fibers. The best result is obtained with a petroleum-based wax, but also the bio-based waxes lead to good mechanical properties of the biocomposite. Thus, the addition to PLA of potato pulp powder, treated with waxes, appears a method able to (i) utilize and valorize an abundant agro-food biomass such as potato pulp, according to the principles of circular economy, (ii) favor the production of articles with properties valuable for practical applications, and (iii) reduce the cost of the final products, considering the relatively high cost of PLA.

## 1. Introduction

Biocomposites are a special class of composite materials, obtained by blending natural fibers with bio-based and/or biodegradable polymers (biopolymers). Biocomposites represent an ecological and low-cost alternative to conventional petroleum-derived materials, and for this reason are becoming progressively more utilized for a wide variety of uses. The interest in the realization of biocomposites is growing rapidly and continuously, as attested by many reviews and books recently published [1,2,3,4,5,6,7]. With respect to conventional composites, in which synthetic fibers, like ceramic or glass fibers, and traditional fillers, like mica, are used, biocomposites offer a wide variety of advantages, such as renewability and low density, which make them lightweight and economical materials. In addition, if the polymeric matrix is biodegradable, the total biodegradability of the products is assured.

The chemical composition of the natural fibers derived from plants consists mainly of cellulose (50–70 wt.%), hemicellulose (10–20 wt.%), lignin (10–30 wt.%), and pectin and waxes in smaller amounts [2,6]. Also, the physico-mechanical properties of the natural fibers depend on the original plants. The density, elastic modulus, tensile strength and elongation at break are approximately in the ranges 0.8–1.5 g/cm^3^, 5–20 GPa, 200–900 MPa, and 1.5–20%, respectively [2,6,7].

The marked hydrophilic nature of natural fibers represents a great problem for the realization of biocomposites with improved properties, due to the general poor compatibility with hydrophobic or less hydrophilic polymer matrices. This scarce compatibility can be improved by modifying the fiber surface properties [1,2,5,6]. Physical and chemical methods can be utilized. Some physical treatments, as for example stretching or calandering, can enhance the interface polymer/natural fibers, without changing the chemical composition of the fibers. Plasma or corona treatment can induce compatibilization between hydrophilic fibers and hydrophobic matrix, through formation of free radicals and surface cross-linking. On the other hand, chemical methods utilize additional materials with intermediate properties as coupling agents. Simple coatings can be used to reduce the hydrophilicity of the lignocellulosic fibers, and thus modify the surface composition. In addition, chemical bonds between the fibers and the matrix can be produced by chemical treatments, in order to reduce the fiber hydrophilic nature or to increase the surface roughness.

The mechanical properties of a biocomposite results from both the matrix and fiber properties, and strongly depend on the adhesion between the fibers and the matrix, which determines the efficiency of the load transmission from the matrix to the fibers. The tensile strength is strongly influenced by the adhesion, whereas the tensile modulus is more affected by the fibers’ distribution, orientation and aspect ratio (length to width ratio). A strong adhesion leads to enhanced strength and stiffness of the composite [8,9]. Thus, the volume fraction of the fibers and their properties, together with the fiber/matrix adhesion are crucial factors that contribute to the final properties of natural fiber reinforced composites [10]. In a composite, the matrix plays the role of transferring the applied stress to the fibers. This occurs at the interface, in the presence of good matrix/fiber adhesion [11]. Unfortunately, poor adhesion often characterizes biocomposites made of hydrophobic polymers and hydrophilic natural fibers. In addition, natural fibers tend to form aggregates during processing, and their hydrophilic nature provokes absorption of moisture, in an amount that can vary between 5% and 10%, with the result that the process ability is worsened. The fiber aspect ratio strongly influences the tensile modulus and the fracture properties, and a critical fiber length is necessary to develop high stiffness and strength of the composite. Fibers with low aspect ratio and not regular shape behave as fillers, and not as reinforcement.

Organic wastes have been sometimes utilized as reinforcement or additives for various polymers [12]. As significant amounts of organic wastes from industry and agriculture remain unutilized, it follows that the use of organic residue materials in biocomposites can represent an ecologically friendly method to produce materials for different applications. This can also allow to reduce the cost of the final products.

Potato wastes are biomasses rich in starch and lignocellulosic constituents. After extraction of starch, the potato pulp accumulates in high amounts. Within the European Union, about 140,000 tons of dried potato pulps are produced annually in the starch industry [13]. The original potato pulp contains water up to about 90%, but drying processes can increase the dry matter up to about 90 wt.%. Dried potato pulp, which consists mainly of lignocellulosic fibers, starch and, at a lesser extent, proteins, can be used as filler or reinforcement for biopolymers. The cost of this raw material is low, which makes potato pulp even more interesting for industrial utilization [14].

Poly(lactic acid) (PLA) is the bio-based and biodegradable polymer most present on the market, widely used for packaging purposes. Many properties of PLA, such as strength and stiffness are comparable to those of traditional petroleum-based polymers, whereas a relatively low toughness limit its use [15]. Several types of natural fibers have been used to produce PLA based biocomposites [16,17,18,19,20], for which the effects of the processing conditions and/or surface chemical modification of the fibers on the mechanical, thermal and viscoelastic properties have been investigated. Recently also potato pulp powder has been utilized to produce biocomposites with PLA [21].

In the present study, biocomposites made of PLA and 20 wt.% of potato pulp powder (PPP) have been produced by melt mixing, processed by injection extrusion and characterized in terms of thermal, mechanical and viscoelastic properties. A previous article on the effect of increasing percentage of PPP (5, 10 and 20 wt.%) added to PLA, demonstrated that PPP can be mixed with PLA and easily processed up to 20 wt.% [21], with the addition of a non-toxic plasticizer, acetyl-tri-n-butyl citrate (ATBC) [22] and calcium carbonate (CaCO_3_), which acts as inert filler to facilitate the removal injection molded specimen from the mold. In an attempt to improve the adhesion between PLA and PPP, in the present study potato pulp fibers coating with bio-based and petroleum-based waxes was investigated.

## 2. Materials and Methods

### 2.1. Materials

Poly(lactic acid) (PLA) derived from natural resources, was 2003D NatureWorks (Minnetonka, MN, USA), grade for thermoforming and extrusion processes, which contains 3% of d-lactic acid units melt flow index (MFI): 6 g/10 min (210 °C, 2.16 kg), nominal average molar mass: 200,000 g/mol.

The plasticizer acetyl-tri-n-butyl citrate (ATBC) was purchased from Sigma Aldrich S.R.L (Milan, Italy).

The calcium carbonate (CaCO_3_) OMYACARB^®^ was an inert filler supplied by the company OMYA (Oftringen, Switzerland). The powder, which has fine granulometry, with particle size distribution centered at 12 μm, is used to facilitate the removal injection molded specimen from the mold. 

The dried potato pulp powder (PPP) was produced by the company (SüdStärke, Schrobenhausen, Germany). The moisture content was about 3 wt.%, and the composition of the dry matter: proteins 7 wt.%, starch 25 wt.%, cellulose 16 wt.%, hemicellulose 7 wt.%, lignin 20 wt.%, pectin 17 wt.%, ash 5 wt.%.

Commercial wax-based additives Aquacer 561, Aquacer 581, and Aquacer 593 were provided by BYK Additives & Instruments (Wesel, Germany). They are (i) non-ionic aqueous emulsions of bee wax (Aquacer 591), (ii) non-ionic aqueous emulsions of carnauba wax (Aquacer 581), and (iii) non-ionic aqueous emulsion of a modified polypropylene wax (Aquacer 593).

### 2.2. Composite Preparation

PLA based biocomposites were prepared by adding 20 wt.% of potato pulp powder to a polymeric matrix, constituted by the biopolymer PLA, with concentration 85 wt.%, the plasticizer ATBC, with concentration 10 wt.%, and CaCO_3_, with concentration 5 wt.%.

For the preparation of the PLA based biocomposites with potato pulp powder coated with waxes, 20 mL of wax aqueous emulsion with concentration 5% w/v were added to 19 g of PP powder. The mixture was carefully hand blended for sufficient long time. Before processing, PLA and the potato pulp powder, non-coated and coated, were dried at a temperature of 60 °C for at least 24 h.

The PLA based matrix and biocomposites were prepared by using a MiniLab II HAAKE Rheomex CTW 5 (Waltham, MA, USA), a co-rotating conical twin-screw extruder. The molten materials were transferred from the mini extruder through a preheated cylinder to a mini injection molder (Thermo Scientific HAAKE MiniJet II) (Waltham, MA, USA), which allows to prepare dog-bone tensile bars specimens, to be used for thermal, mechanical and viscoelastic characterization. The dimensions of the dog-bone tensile bars were: width in the larger section: 10 mm, width in the narrow section: 4.8 mm, thickness 1.35 mm, length 90 mm. The extruder operating conditions adopted for all the formulations are reported in Table 1. After preparation, all the samples were stored in a desiccator and analyzed the day after in order to avoid physical ageing effects on the physical properties. The PLA based samples investigated in the present study, with the relative composition, are listed in Table 2. In the biocomposites, the weight percentage of the matrix [PLA(85 wt.%) + ATBC(10 wt.%) + CaCO_3_(5 wt.%)] was maintained constant (80 wt.%), and the PPP amount was reduced to 19 wt.% in the presence of 1 wt.% of waxes.

### 2.3. Composite Characterization

The thermal stability of the potato pulp powder and selected samples was investigated by thermogravimetric analysis (TGA) carried out on about 10 mg of sample by using a Perkin Elmer TGA 7 (Waltham, MA, USA), under nitrogen flow (35 mL/min), at a heating speed of 10 K/min from 50 °C to 600 °C.

Number-average molar mass (*M_n_*) and weight-average molar mass (*M_w_*) were determined using size exclusion chromatography (SEC), Agilent Technologies 1200 Series (Santa Clara, CA, USA) and calculated with the Agilent ChemStation Software. The instrument was equipped with an Agilent degasser, an isocratic HPLC pump, two PLgel 5 μm MiniMIX-D columns conditioned at 35 °C and an Agilent refractive index (RI) detector. The mobile phase was chloroform (CHCl_3_) at a flow rate of 0.3 mL/min. The system was calibrated with polystyrene standards in a range from 500 to 3 × 10^5^ g/mol. Samples were dissolved in CHCl_3_ (2 mg/mL) and filtered through a 0.20 μm syringe filter before analysis.

Differential scanning calorimetry (DSC) measurements were performed with a Perkin Elmer Calorimeter DSC 8500 (Waltham, MA, USA), equipped with an IntraCooler III as refrigerating system. The instrument was calibrated in temperature with high purity standards (indium, naphthalene, cyclohexane) according to the procedure for standard DSC [23]. Energy calibration was performed with indium. Dry nitrogen was used as purge gas at a rate of 30 mL/min. The as prepared PLA based matrix and biocomposites were analyzed from −50 °C to 200 °C at the heating rate of 10 K/min.

Tensile tests on the PLA based matrix and biocomposites prepared with the injection molder were performed at room temperature, at a crosshead speed of 10 mm/min, by means of an INSTRON 5500 R universal testing machine (Canton, MA, USA), equipped with a 10 kN load cell and interfaced with a computer running the Testworks 4.0 software (MTS Systems Corporation, Eden Prairie, MN, USA). At least five specimens were tested for each sample in according to the ASTM D 638, and the average values reported.

Oscillatory shear measurements were performed by means of a Rheometer Anton Paar MCR 302 (Graz, Austria), equipped with parallel plates of 25 mm diameter, under nitrogen flow to minimize oxidation and to maintain dry environment. Frequency sweep experiments were performed at 175 °C at a fixed strain (3%), with gap of 1 mm, in the linear regime, in order to measure the modulus of the complex viscosity |η*| and the storage and loss moduli, G’ and G”, respectively. The analysis of the PLA based matrix and biocomposites was performed in the narrow angular frequency range from 3.14 to 12.6 rad/s, to reduce the time measurement and avoid degradation of the potato pulp.

The morphology of the potato pulp powder and PLA based matrix and biocomposites was investigated by scanning electron microscopy (SEM) with an FEG-Quanta 450 ESEM instrument (Waltham, MA, USA). The micrographs of samples fractured with liquid nitrogen and etched with gold were collected. Backscattered electrons generated the images whose resolution was provided by beam deceleration with a landing energy of 2 kV.

## 3. Results and Discussion

The thermal, mechanical and viscoelastic properties of the PLA based matrix and biocomposites with non-treated and treated potato pulp powder were investigated in order to quantify how (i) the addition of these fibers changes the structure of the polymeric material, and (ii) the treatment of the potato pulp powder with waxes modifies the polymer/filler adhesion. These data together provide information on possible miscibility/compatibility between PLA and the non-treated and treated potato pulp powder.

### 3.1. Thermogravimetric Analysis of the Potato Pulp Powder and PLA Matrix and Biocomposites

**Figure 1:**
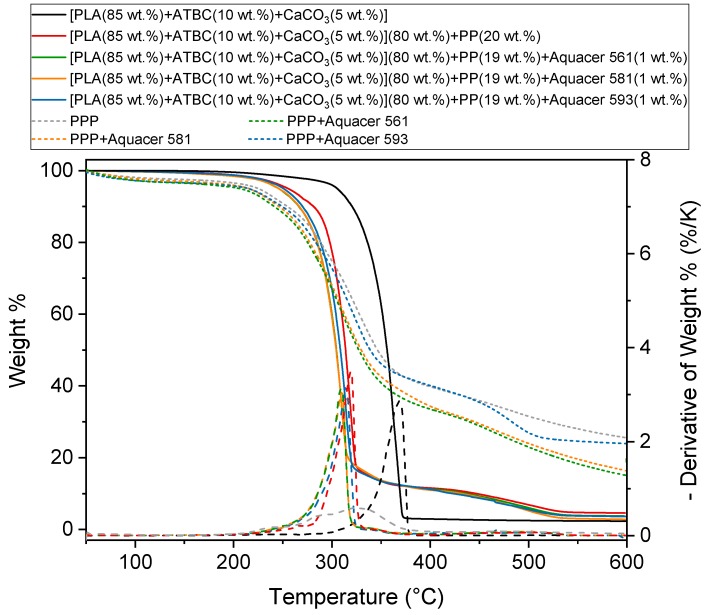
Thermogravimetric curves of the uncoated and coated potato pulp powder (PPP), and the poly(lactic acid) (PLA) based matrix and biocomposites indicated in the legend at 10 K/min under nitrogen flow (estimated error: ± 0.2 wt.%). The dotted lines are the derivative of the wt.% curves.

The thermal stability of the potato pulp powder and the PLA based biocomposites without and with wax coating was determined by means of the thermogravimetric analysis under nitrogen flow, because the contact of the material with air is reduced in the extruder and moulder. Figure 1 shows the thermogravimetric curve of the potato pulp powder without and with coating with waxes. The initial weight loss detected at temperatures lower than 150 °C is due to water vaporization. The water content in coated and uncoated PPP is approximately 3 wt.%. The weight residue that is observed at high temperature is due to the carbon deposit that remains in the presence of an inert atmosphere. The thermal degradation of natural fibers is generally a multiple process, due to the contribution of the main different components, i.e., cellulose, hemicellulose and lignin. The maximum weight loss of natural fibers generally occurs in the temperature interval 200–350 °C, with a solid residue at 600 °C in the range 20–40% [24,25]. Starch degradation extends from approximately 300 °C to 350 °C, and its solid residue at 600 °C is approximately 20% [26]. Also, degradation of proteins occurs in similar temperature range, between 200 °C and 400 °C, with maximum loss rate around 300 °C, and a 20% residue at high temperatures [27]. From Figure 1 the potato pulp powder appears stable up to about 190 °C. This thermal stability assures that the potato pulp powder does not undergo substantial degradation during the processing of the PLA biocomposites at 180 °C, being the residence time at this temperature not longer than 1.5 min.

Figure 1 shows also the thermogravimetric curves of the PLA based matrix and biocomposite with 20 wt.% of PPP without and with surface treatment of the potato pulp with the waxes. The thermal degradation of the PLA based matrix occurs in a single step in a quite narrow temperature range. The initial degradation temperature of the PLA based matrix is located at about 290 °C, whereas the maximum degradation rate is centered at about 370 °C, in excellent agreement with previous studies [28,29,30]. 

The thermal degradation of the biocomposites takes place in multiple steps, as cumulative process of the matrix and the filler. Biocomposite with coated and uncoated PPP starts to degrade at about 190 °C. The maximum degradation rate of the biocomposites with untreated PPP shifts to around 320 °C, and to slightly lower temperatures in the presence of PPP treated with waxes.

In order to investigate if the lower thermal stability of the biocomposites has to be ascribed to reduced molar mass, as a consequence of PLA degradation occurring during the processing at 180 °C in the presence of the moisture introduced by PPP [31], the number-average molar mass (*M_n_*) and the mass-average molar mass (*M_w_*) of PLA in the matrix and biocomposites were measured by size exclusion chromatography (SEC). Table 3 shows that the PLA molar mass in the biocomposite with PPP not treated with waxes is lower than that of the PLA matrix, but the molar mass decreases more markedly in the presence of PPP treated with waxes, probably due additional degradation processes induced by the chemical components of the waxes.

From data reported in the literature on the effect of the molar mass on the thermal stability of PLA, it can be deduced that the marked decrease in thermal stability of the biocomposite with uncoated PPP cannot be ascribed exclusively to the lower molar mass, because the reduction in the degradation temperature should be not higher than 10 K [32]. Thus, the temperature reduction of the main degradation step of biocomposites with respect to the PLA matrix has to be ascribed to combined degradation process of the matrix and the natural fibers. For PLA, hydrolytic breakage of ester linkage by water molecules, depolymerisation, cyclic oligomerization, and transesterification reactions have been reported as possible degradation mechanisms [28,33]. On the other hand, the shift of the degradation process towards lower temperatures for the biocomposites with coated PPP could be associated with the reduction in PLA molar mass, as well as to likely accelerate degradation reactions caused by the waxes’ components.

### 3.2. Thermal Properties of the PLA Matrix and Biocomposites

**Figure 2:**
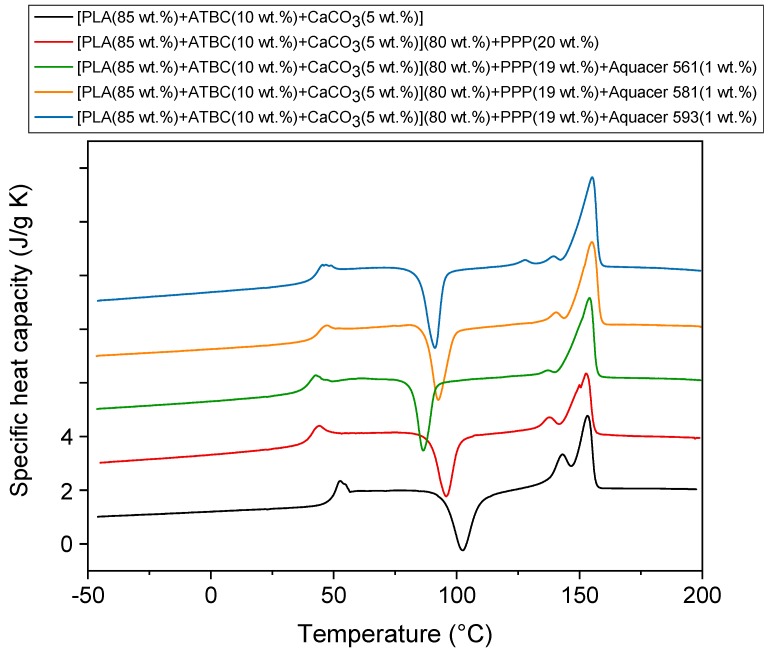
Specific heat capacity (*c_p_*) of the as prepared PLA based matrix and biocomposites indicated in the legend, as a function of the temperature. The curves were measured upon heating at 10 K/min after previous fast cooling to −50 °C. The ordinate values refer only to the bottom curve. All the other curves are shifted vertically for the sake of clearness.

The specific heat capacity (*c_p_*) curves of the as prepared PLA based matrix and biocomposites with potato pulp powder non-treated and treated with the waxes, measured at 10 K/min after rapid cooling down to −50 °C, are shown in Figure 2. As described in the section Materials and Methods, all the samples were processed for 1 min at 90 °C. The main melting process of the waxes, which is not visible in the *c_p_* curves due to their small amount, is located at approximately 65 °C, 85 °C, and 160 °C for Aquacer 561, Aquacer 581, and Aquacer 593, respectively.

The glass transition, which occurs in proximity of 60 °C, in agreement with literature data [34], is overlapped by an enthalpy recovery peak, due to permanence of the samples at room temperature for one day. Before the melting endotherm, all the curves display an intense cold crystallization peak located approximately in the interval between 90 °C and 110 °C for the PLA based matrix and between 80 °C and 100 °C for the biocomposites. At temperatures lower than 100 °C, the crystal α′-form of PLA mainly grows, whereas in the temperature range from 100 °C and 120 °C, a mixture of α′- and α-form develops [35]. At the heating rate of 10 K/min, the α′-crystals transform into the more ordered α-phase via melting and almost simultaneous recrystallization [34]. As a consequence, the melting behavior that extends from approximately 130 °C to 160 °C, results from the fusion of both the α′- and the α-crystals. Reorganization and recrystallization events overlap the entire fusion process, as generally takes place in semi-crystalline polymers at a relatively low heating rate [36,37,38,39].

Table 4 lists the *T_g_* values together the enthalpy of cold crystallization (Δ*h_c_*) and the enthalpy of fusion (Δ*h_m_*) of the samples investigated, calculated from the *c_p_* curves shown in Figure 2. The addition of PPP affects the glass transition temperature of PLA. The *T_g_* value of the biocomposite not containing waxes is lower than that of the polymeric matrix, which could be caused by weak interactions between PLA and the fibers, with consequent formation of free volume at the matrix/filler interface. Conversely, *T_g_* is found to increase after treatment of the potato pulp powder with Aquacer 581, and Aquacer 593, which attests the establishment of interactions between the polymeric matrix and the fibers, with the result that the molecular mobility of the polymeric chains is reduced. This finding proves the efficiency of Aquacer 581, and Aquacer 593, as coating agents for the PLA based biocomposites with potato pulp powder.

The measured Δ*h_c_* and Δ*h_m_* values collected in Table 4 were normalized to the PLA content [40]. From these experimental values, an estimation of the crystalline weight fraction growing during the cold crystallization process (*w_Cc_*), and disappearing during the melting process (*w_Cm_*) was thus obtained, dividing Δ*h_c_* and Δ*h_m_* by the enthalpy of fusion of 100% crystalline PLA phase (Δ*h_m_*) at the crystallization and melting temperatures respectively. For biocomposites, cold crystallization occurs almost completely at temperatures lower than 100 °C, which means that only α′-form develops [35]. At the heating rate of 10 K/min, however these α′-crystals are expected to transform into the more ordered α-phase [36]. For biocomposites, the crystal weight fraction *w_Cc_* was calculated by dividing the measured Δ*h_c_* by Δ*h_m_* for the α′-form, i.e., 81 J/g for the cold crystallization centered at about 95 °C or 78 J/g for the cold crystallization centered at about 90 °C, whereas *w_Cm_* was obtained from the ratio between Δ*h_m_* and the enthalpy of fusion of the α′-form at about 150 °C (Δ*h_m_* =108 J/g) [41], as the enthalpy of crystallization and the enthalpy of fusion of the α-crystals cancel out. For the PLA based matrix, for which cold crystallization extends up to 115 °C, with simultaneous growth of α′- and α-crystals, the average values between the enthalpy of fusion of the α′- and α-forms were utilized, i.e., Δ*h_m_* =96 J/g for the cold crystallization centered at about 100 °C, and Δ*h_m_* =119 J/g for the melting process centered approximately at 150 °C [41].

The *w_Cc_* and *w_Cm_* values listed in Table 4 reveal that PLA in the matrix and biocomposites is completely amorphous after processing for 1 min at 90 °C. This can be explained by considering the small time that characterize the injection molding process and the slow crystallization kinetics of PLA [42]. The peak temperature of the cold crystallization process decreases in the biocomposites with respect to the PLA matrix, due to the lower mass and likely nucleating action exerted by the potato pulp powder. In this case, the formation of a transcrystalline layer around the fibers has been described [43,44]. Transcrystalline morphology is characterized by a high density of nucleating crystallites, which grow with an orientation perpendicular to the surface responsible for nucleation [45]. The reduction in the cold crystallization temperature peak is higher after addition of Aquacer 561, which proves the higher nucleating effect of the potato pulp particles coated with bee wax. However, the marked nucleating action exerted by the coated potato pulp powder on PLA is attested also by the higher crystallinity that develops during cold crystallization.

### 3.3. Mechanical Properties of the PLA Matrix and Biocomposites

**Figure 3:**
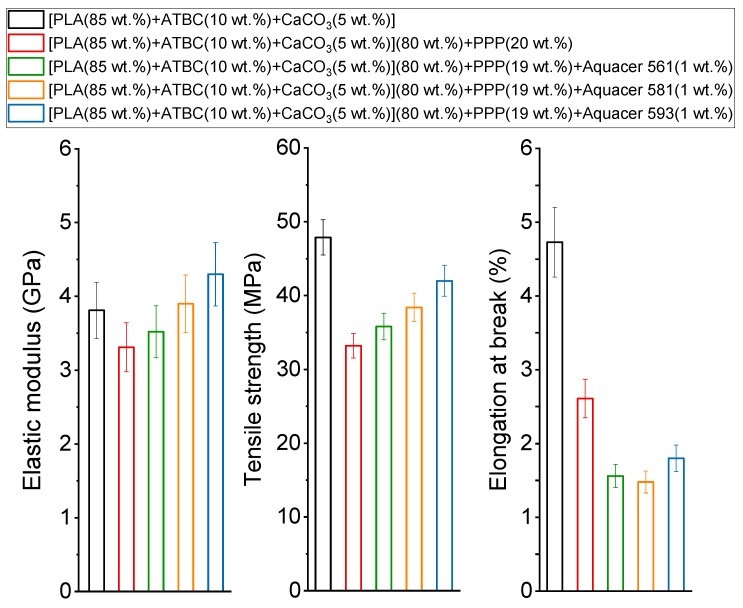
Elastic modulus, tensile strength and elongation at break of the PLA base matrix and biocomposites indicated in the legend.

The mechanical properties of the PLA based matrix and biocomposites with PPP non-treated and treated with the bio-based and petroleum-based waxes are summarized in Figure 3. The tensile modulus was obtained from the slope of the initial stress vs. strain curve, whereas the tensile strength was the maximum stress value or ultimate stress. The tensile elastic modulus of the biocomposite containing non-treated potato pulp powder was found smaller with respect to the PLA matrix. As for *M_w_* higher than about 100,000 g/mol the mechanical properties of PLA are not influenced significantly by molar mass [46], the decrease in the elastic modulus has to be ascribed to the action of PPP as filler, and not as reinforcement for PLA. As a general rule, the elastic modulus of a composite increases with increasing the fiber content, if the rigidity of the filler is higher than that of the polymeric matrix and if the length of the filler is sufficiently high [47,48]. Thus, the behavior displayed by the PLA based biocomposites with PPP has to be ascribed to the low aspect ratio of the potato pulp particles and their irregular shape [21]. Poor adhesion between the potato pulp powder and the polymeric matrix is attested by the lower tensile strength exhibited by the biocomposite. Potato pulp powder is composed mainly by lignocellulosic fibers and starch, which are highly hydrophilic, so that poor interactions are expected with less hydrophilic polymers. The immiscibility between PLA and starch has been indeed demonstrated in many studies [3,49]. Also, the elongation at break of the biocomposite appears smaller than that of the polymeric matrix, because the dispersed filler particles act as stress concentrators, which leads to reduced ductility of the material. Under stress, this situation promotes microcrack formation at the polymer/filler interface.

Figure 3 reveals that the treatment of the potato pulp powder with Aquacer 561, Aquacer 581, and Aquacer 593 improves the mechanical properties of the PLA biocomposites, because the elastic modulus is found to increase with respect to the biocomposite with untreated filler. The tensile elastic modulus of the biocomposite with potato pulp powder coated with Aquacer 593 is even higher than that of the PLA matrix, which attests that in this case the potato pulp particles act as reinforcement for the polymeric matrix, due to an improved adhesion polymer/filler. This is confirmed also by the tensile strength, which increases in the order Aquacer 561 < Aquacer 581 < Aquacer 593. The finding demonstrates that the matrix/fiber adhesion improves and that better interfacial interactions are established between the polymer matrix and the potato pulp particles, which become less hydrophilic by treatment with the waxes Aquacer 561, Aquacer 581 and Aquacer 593. The better adhesion between PLA and the potato pulp particles treated with Aquacer 561 and Aquacer 581 can be ascribed to the composition of the bio-based waxes. Both carnauba and bee waxes are rich in C_16_–C_50_ esters and hydroxyl esters (approximately 50% and 15% in constituent composition, respectively) [50,51], which can favor interactions with the polymeric matrix and the hydrophilic potato pulp powder, respectively. Unfortunately, the detailed composition of the commercial Aquacer 593 is unknown. The increased interaction results in an improved load and stress transfer between the polymeric matrix and the filler. Conversely, the addition of potato pulp powder to the polymeric matrix reduces the ductility of the biocomposites also after treatment with the waxes.

To summarize, the biocomposite containing untreated potato pulp powder exhibits worse mechanical properties with respect to the PLA based matrix, but the surface treatment of the potato pulp particles with Aquacer 561, Aquacer 581, and Aquacer 593 improves the elastic modulus and tensile strength of the biocomposite.

In order to quantify the matrix/filler adhesion in composites, the values of the elastic modulus can be compared with theoretical predictions according to analytical models [52,53,54]. These models generally contain parameters that depend on the matrix-filler interaction and/or shape and distribution of the particles. Sato and Furukawa [52] developed a model that correlates the tensile modulus of a composite with an adhesion parameter (ξ), which ranges from 1 (poor adhesion) and 0 (perfect adhesion). The analytical equation is:

(1) $${E=E}_{m}\left[\left(1+\frac{0.5\phi^{2/3}}{1-\phi^{1/3}}\right)\right]\left(1-\psi \xi \right)-\frac{\phi^{2/3}\psi \xi }{\left(1-\phi^{1/3}\right)\phi }$$

with

(2) $$\psi =\left(\frac{\phi }{3}\right)\left[\frac{1+\phi^{1/3}-\phi^{2/3}}{1-\phi^{1/3}+\phi^{2/3}}\right]$$

where E_m_ is the tensile modulus of the matrix and Φ the volume fraction of the filler, which was calculated by using as density of the amorphous PLA the value 1.248 g/cm^3^ [55], and as density of the potato pulp powder, the measured value 0.35 g/cm^3^. The calculated adhesion parameter ξ for the biocomposites with potato pulp powder treated and non-treated with the waxes are listed in Table 5, together with the elastic modulus E, in order of increasing E value. A very good agreement between the trend of the elastic modulus and the trend of the adhesion parameter, which decreases with improving the adhesion, can be observed. This confirms that the transfer of the applied stress from the matrix to the potato pulp particles, which occurs at the polymeric matrix/filler interface, is favored by good adhesion, according also to the trend of the tensile strength (Figure 3).

### 3.4. Viscoelatic Properties of the PLA Matrix and Biocomposites

The study of the viscoelastic properties is crucial to gain a fundamental understanding of the processability of a polymeric material, especially for injection molding and extrusion processes. In addition, for a mixture, it provides information on the state of dispersion and adhesion of the components. The analysis was performed under dynamic conditions, because from oscillatory measurements details on both the elastic and viscous properties can be obtained [56]. Dynamic oscillatory shear measurements of polymeric materials are generally performed by applying a time dependent strain γ(*t*) = γ_0_·sin(ω*t*), and measuring the resultant shear stress σ(t) = σ_0_·[G′sin(ω*t*) + G″cos(ω*t*)], where G′ and G″ are the storage and loss moduli, respectively and ω the angular frequency. In addition, the modulus of the complex viscosity |η*|can be derived, as it holds that |η*|(ω) = [G′(ω)^2^ + G″ (ω)^2^]^1/2^/ω [56].

**Figure 4:**
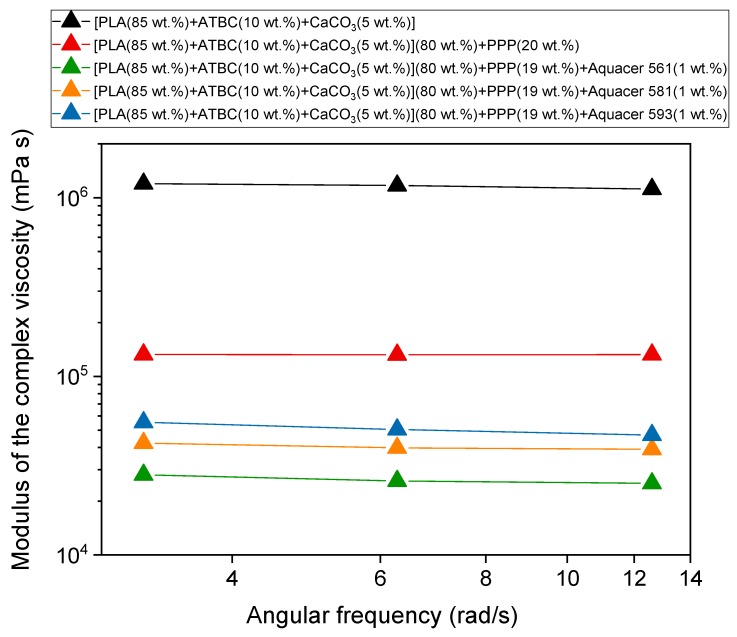
Modulus of the complex viscosity (|η*|) *vs*. angular frequency (ω) at 175 °C for the PLA based matrix and biocomposites indicated in the legend. The solid lines are a guide to the eye.

The bilogarithmic plot of |η*| vs. ω, obtained by parallel plate oscillating rheometer at 175 °C for PLA based matrix and biocomposites with potato pulp powder non-treated and treated with the bio-based and petroleum-based waxes, is shown in Figure 4. The experiments were performed from high to low frequencies, in a narrow deformation frequency range outside but close to the Newtonian plateau [21], in order to reduce considerably the measurement times, and, consequently, the fibers degradation. In general, PLA behaves like a pseudo-plastic, non-Newtonian fluid and a typical shear thinning fluid, in which at high shear rates the macromolecules orient and the entanglements number decreases [57,58].

Figure 4 reveals that the viscosity of the biocomposites is smaller than that of the PLA polymeric matrix. As previously discussed [21], by considering the lower PLA molar mass of the biocomposite with untreated 20 wt.% of PPP with respect to the matrix, the decrease in the modulus of the complex viscosity is higher than that expected according to the relationship: log η_o_ = logK + 3.4log*M_w_* (with logK = −11.7), where η_o_ is the zero shear viscosity referring to the Newtonian plateau [57,58]. This result proves the presence of free volume at the polymer-fibers interfaces, connected to poor adhesion between potato pulp powder and PLA, which favors the flow of the PLA chains. Also, for the biocomposites with PPP coated with waxes, characterized by lower *M_w_* values, the decrease in viscosity is higher than the one predicted by the above relationship. The finding confirms that also the biocomposites with coated PPP exhibit no strong polymer/filler adhesion, although better with respect to untreated PPP, as also proven by the tensile strength trend (Figure 3). It is worth noting that the viscosity increases with increasing the interfacial interaction, i.e., to mention the waxes, in the order Aquacer 561 < Aquacer 581 < Aquacer 593.

**Figure 5:**
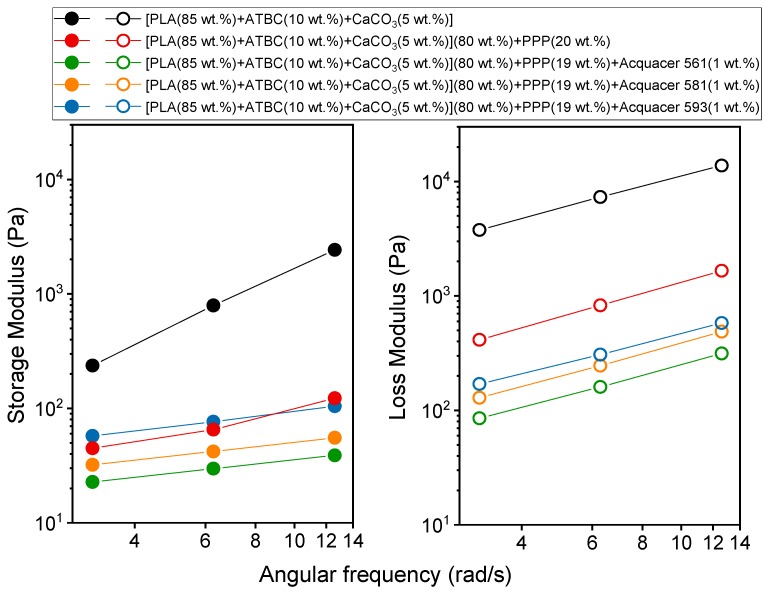
Storage and loss moduli (G′ and G″, respectively) vs. angular frequency (ω) at 175 °C for the PLA based matrix and biocomposites indicated in the legend. The solid lines are a guide to the eye.

The storage and loss moduli (G′ and G″) of PLA based matrix and biocomposites with potato pulp powder non-treated and treated with the bio-based and petroleum-based waxes are shown in Figure 5. The slopes of G′ vs. ω for the biocomposites with potato pulp treated with waxes are slightly lower with respect to the biocomposite with non-coated potato pulp and the PLA based matrix. This suggests a tendency towards a solid-like behavior of the biocomposites with the potato natural fibers treated with waxes.

At 175 °C, the dependence of the storage modulus on the samples composition appears different from the one observed for the tensile elastic modulus in the solid state (Figure 3), because G’ of the PLA matrix exhibits the highest values This can be ascribed to the effect of the temperature: with increasing the temperature, the intermolecular interactions become weaker, because of the increasing Brownian motions that progressively destroy or loosen the intermolecular bonds. The result is that the filler/matrix adhesion worsens, so that at 175 °C, for the angular frequency investigated, the storage modulus of the biocomposites is smaller than that of the PLA based polymeric matrix, with the exception of the biocomposite with potato pulp treated with Aquecer 593. Thus, also Figure 5 (left) proves that stronger and more resistant interactions are established between the PLA based matrix and the potato pulp particles coated with Aquacer 593.

In regard to the loss modulus, Figure 5 (right) shows that the dependence of G” on the biocomposite composition appears similar to that of the viscosity, because it represents the viscous part of the stress, i.e. the part of the stress where energy is dissipated.

### 3.5. Morphological Properties of the PLA Matrix and Biocomposites

The morphology of fracture surfaces of PLA based matrices and biocomposites from dog-bone specimens was studied by scanning electron microscopy, in order to investigate the dispersion of the potato pulp particles and the compatibility between the matrices and the fibers. Data of each single scanning are reported in the legend of each specific image.

**Figure 6:**
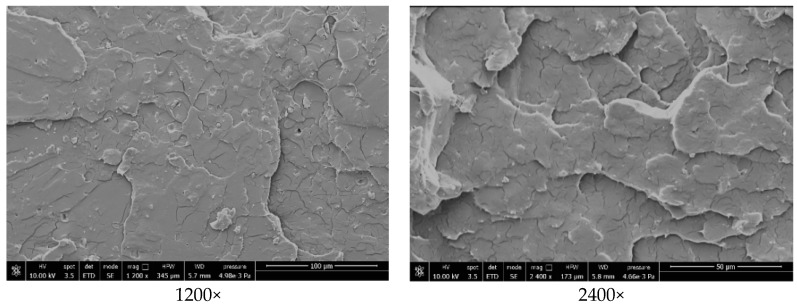
SEM images of the PLA based matrix at the indicated magnification.

**Figure 7:**
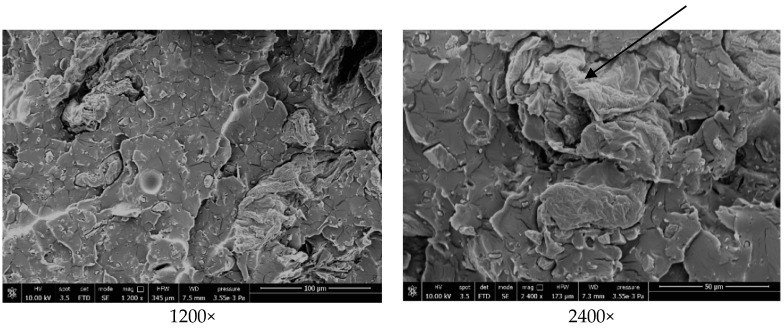
SEM images of the [PLA(85 wt.%) + ATBC(10 wt.%) + CaCO3(5 wt.%)](80 wt.%) + PPP(20 wt.%) biocomposite at the indicated magnification.

Figure 6 illustrates the topology of the pure PLA matrix, which appears smooth and without evident voids. The surfaces of the PLA based biocomposites containing 20 wt.% of potato pulp powder are shown in Figure 7. The potato pulp particles appear well dispersed within the matrix and their distribution uniform, which means that they were satisfactorily separated during the extrusion process. The micrographs clearly show that the interfacial adhesion between the PLA matrix and the potato pulp powder is quite poor, because a gap is well evident between the polymeric matrix and the PP particles. This leads to brittle materials, as also proven by the fiber pullout indicated by an arrow, which illustrates how in this biocomposite failure occurs at the matrix/fiber interface. The finding is in agreement with the decrease in the tensile strength observed after addition of the potato pulp powder to the PLA matrix (Figure 3).

**Figure 8:**
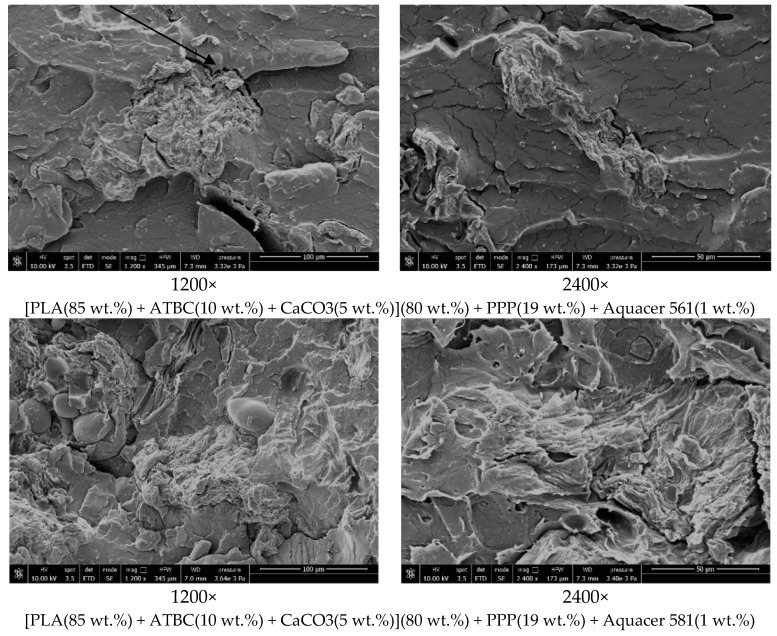
SEM images of the [PLA(85 wt.%) + ATBC(10 wt.%) + CaCO3(5 wt.%)](80 wt.%) + PPP(19 wt.%) + Aquacer 561(1 wt.%) and [PLA(85 wt.%) + ATBC(10 wt.%) + CaCO3(5 wt.%)](80 wt.%) + PPP(19 wt.%) + Aquacer 581(1 wt.%) biocomposites at the indicated magnification.

SEM observations on the PLA based biocomposite after fiber surface treatment with the bio-based waxes Aquacer 561 and Aquacer 581 indicate differences with respect to the untreated biocomposites (Figure 8). After surface treatment of the potato pulp particles, the fracture surface of the PLA based biocomposite appears locally continuous and smoother, indicating that the compatibility of the matrix and the fibers is improved. Some bridging effect can be observed between the PLA matrix and the fibers, as indicated by the arrow. Bridging effect can prevent crack propagation and enable effective stress transfer between the matrix and the fibers, leading to better mechanical tensile properties, as previously demonstrated and discussed. The round shaped particles detected at 1200× magnification in the PP powder are either starch or pectin, because they disappear after treatment with amylase and pectina. Thus, the surface treatment of the potato pulp powder with the bio-based waxes Aquacer 561 and Aquacer 581 is proven to improve wetting with the PLA matrix, which results in a better fiber-PLA adhesion with respect to the untreated fiber-PLA matrix system.

## 4. Conclusions

Biocomposites made of PLA and 20 wt.% of potato pulp powder have been produced by melt mixing, processed by injection extrusion and characterized in terms of thermal, mechanical and viscoelastic properties. Potato pulp powder acts as filler, and not as reinforcement for the PLA based matrix, because a decrease in elastic modulus, tensile strength and elongation at break is produced. This behavior can be ascribed to the unfavorable aspect ratio of the potato pulp particles, which present short length and not regular shape, and to poor matrix/filler adhesion.

In an attempt to improve the interaction between PLA and potato pulp powder and reduce the tensions at the interface, in the present study fiber coating with bio-based and petroleum-based waxes was employed and investigated. The wax content in the final products was 1 wt.%.

The surface treatment of the fibers with waxes was found to improve the mechanical properties of the biocomposites, enhancing the adhesion between the PLA based matrix and the potato pulp fibers. The best results were obtained with a petroleum-based wax, but also the bio-based waxes led to good mechanical properties of the biocomposite.

Thus, the addition to PLA of potato pulp powder, treated with waxes, appears a method able to (i) utilize and valorize an abundant agro-food biomass such as potato pulp, according to the principles of circular economy, (ii) favor the production of articles with properties valuable for practical applications, and (iii) reduce the cost of the final products, considering the relatively high cost of PLA.

## Figures and Tables

**Table 1:** Operating condition use for the extrusion and injection molding process.

Extrusion Temperature (°C)	Screw Speed (rpm)	Cycle Time (s)	Injection Temperature (°C)	Injection Pressure (bar)	Molding Time (s)	Mold Temperature (°C)
180	100	90	180	500	60	90

**Table 2:** Composition of the PLA matrix and biocomposites.

Matrix	Potato Pulp Powder	Wax
[PLA(85 wt.%) + ATBC(10 wt.%) + CaCO_3_(5 wt.%)]	-	-
[PLA(85 wt.%) + ATBC(10 wt.%) + CaCO_3_(5 wt.%)](80 wt.%)	PPP(20 wt.%)	-
[PLA(85 wt.%) + ATBC(10 wt.%) + CaCO_3_(5 wt.%)](80 wt.%)	PPP(19 wt.%)	Aquacer 561 (1 wt.%)
[PLA(85 wt.%) + ATBC(10 wt.%) + CaCO_3_(5 wt.%)](80 wt.%)	PPP(19 wt.%)	Aquacer 581 (1 wt.%)
[PLA(85 wt.%) + ATBC(10 wt.%) + CaCO_3_(5 wt.%)](80 wt.%)	PPP(19 wt.%)	Aquacer 593(1 wt.%)

**Table 3:** Number-average molar mass (*M_n_*) and mass-average molar mass (*M_w_*) of PLA in the matrix and biocomposites.

Formulation	*M_n_* (g/mol)	*M_w_* (g/mol)
[PLA(85 wt.%) + ATBC(10 wt.%) + CaCO_3_(5 wt.%)]	92,000	170,000
[PLA(85 wt.%) + ATBC(10 wt.%) + CaCO_3_(5 wt.%)](80 wt.%) + PPP(20 wt.%)	70,000	130,000
[PLA(85 wt.%) + ATBC(10 wt.%) + CaCO_3_(5 wt.%)](80 wt.%) + PPP(19 wt.%) + Aquacer 561(1%)	47,000	76,000
[PLA(85 wt.%) + ATBC(10 wt.%) + CaCO_3_(5 wt.%)](80 wt.%) + PPP(19 wt.%) + Aquacer 581(1%)	54,000	87,000
[PLA(85 wt.%) + ATBC(10 wt.%) + CaCO_3_(5 wt.%)](80 wt.%) + PPP(19 wt.%) + Aquacer 593(1%)	51,000	83,000

**Table 4:** Glass transition temperatures (*T_g_*), enthalpy of cold crystallization (Δ*h_c_*), enthalpy of melting (Δ*h_m_*), and crystalline weight fraction growing during cold crystallization (*w_Cc_*) and disappearing during melting (*w_Cm_*) for the as prepared PLA based matrix and biocomposites. (estimated errors: ±0.5 °C for *T_g_*; ±1 J/g for Δ*h_c_* and Δ*h_m_*, and ±0.02 for *w_Cc_* and *w_Cm_*).

Formulation	*T_g_* (°C)	Δ*h_c_* (J/g)	*w_Cc_*	Δ*h_m_* (J/g)	*w_Cm_*
[PLA(85 wt.%) + ATBC(10 wt.%) + CaCO_3_(5 wt.%)]	50	27	0.28	33	0.28
[PLA(85 wt.%) + ATBC(10 wt.%) + CaCO_3_(5 wt.%)](80 wt.%) + PPP(20 wt.%)	40	28	0.34	37	0.34
[PLA(85 wt.%)+ATBC(10 wt.%) + CaCO_3_(5 wt.%)](80 wt.%) + PPP(19 wt.%) + Aquacer 561(1%)	40	30	0.38	41	0.38
[PLA(85 wt.%) + ATBC(10 wt.%) + CaCO_3_(5 wt.%)](80 wt.%) + PPP(19 wt.%) + Aquacer 581(1%)	43	31	0.38	42	0.39
[PLA(85 wt.%) + ATBC(10 wt.%) + CaCO_3_(5 wt.%)](80 wt.%) + PPP(19 wt.%) + Aquacer 593(1%)	43	31	0.40	42	0.39

**Table 5:** Elastic modulus (E) and calculated adhesion parameter (ξ), according to the Sato and Furukawa model [52].

Formulation	E (GPa)	ξ
[PLA(85 wt.%) + ATBC(10 wt.%) + CaCO_3_(5 wt.%)](80 wt.%) + PP(20 wt.%)	3.3	0.83
[PLA(85 wt.%) + ATBC(10 wt.%) + CaCO_3_(5 wt.%)](80 wt.%) + PPP(19 wt.%) + Aquacer 561(1%)	3.5	0.79
[PLA(85 wt.%) + ATBC(10 wt.%) + CaCO_3_(5 wt.%)](80 wt.%) + PPP(19 wt.%) + Aquacer 581(1%)	3.9	0.74
[PLA(85 wt.%) + ATBC(10 wt.%) + CaCO_3_(5 wt.%)](80 wt.%) + PPP(19 wt.%) + Aquacer 593(1%)	4.3	0.68
